# “Apart From What I Encounter in Clinics” – Medical Students’ Reflective Engagement with Museum Queer Arts

**DOI:** 10.5334/pme.1497

**Published:** 2025-04-09

**Authors:** Krishna Mohan Surapaneni

**Affiliations:** 1 Panimalar Medical College Hospital & Research Institute, IN

## Abstract

**Background & Need for Innovation:**

In contemporary medical education, there remains a notable gap in effectively addressing the complex societal and cultural dimensions of healthcare, particularly regarding the LGBTQIA+ community. Medical students often receive limited exposure to the nuanced aspects of gender and sexual diversity, which is crucial for fostering an inclusive healthcare environment.

**Goal of Innovation:**

This study aimed to bridge this gap by integrating queer museum arts into the undergraduate medical curriculum to promote reflective engagement and dismantling of biases among future healthcare professionals.

**Steps Taken for Development and Implementation of Innovation:**

In this mixed-method study 24 randomly selected final-year medical students underwent a four-week flipped-classroom program divided into three phases: an “Orientation phase” to train students in Visual Thinking Strategies (VTS); an “Exploration” phase, where students engaged with queer arts in small groups to identify and discuss discrimination and bias through a pessimistic lens; and a “Reinforcement” phase, which encouraged reflection on inclusive practices in healthcare through an optimistic perspective. Students were instructed to write a narrative report from a queer perspective in the first phase and a physician perspective in the second phase.

**Evaluation of Innovation:**

Students’ confidence before and after the program were collected and one-on-one semi-structured interviews were conducted. Then, a sequential analysis was performed, using quantitative results to drive qualitative analyses to explore student experiences and evaluate program effectiveness. Engagement with queer arts facilitated deeper emotional and intellectual connections, leading to a transformative shift in perceptions and attitudes towards inclusivity in healthcare. Participants initially exhibited low confidence levels in areas such as gender diversity, gender inequality related discussion, understanding multiple perspectives etc., largely due to fear of judgment, societal stigma, and a lack of prior exposure. However, by the end of the program, significant improvements were observed, with higher confidence across many areas driven by critical reflection and deeper engagement with gender and sexual diversity.

**Critical Reflection on your Process:**

The program served as a catalyst for challenging students to confront their biases through disorienting dilemmas and engage in critical reflection. This deep, internal shift not only broadened their understanding of gender and sexual diversity but also redefined their roles as advocates for inclusivity in healthcare. Program evaluation demonstrated its effectiveness in enhancing awareness, confidence, and equipping future medical professionals with the mindset necessary to create inclusive and compassionate care environments.

## Background and Need for Innovation

Many Medical Education interventions focus on enhancing the procedural and diagnostic skills of students so that they are able to start acquiring the knowledge and skills from the beginning of their medical training [1,2]. This focused approach often leaves behind a paucity of adequate exposure to critical societal issues that has inimical effects on the wider healthcare system and the principles of humanistic medicine [3,4]. Museum art-based education offers an innovative way to address this gap by enhancing students’ observational skills, empathy, and cultural awareness through visual analysis and reflective discussions [5]. While the imperative for procedural and diagnostic expertise is crucial, so is the acquisition of culture and gender-inclusive competencies critical to enhance the delivery of care and patient outcomes [6].

Yet even initiatives supporting the introduction of humanities and inclusivity training [7,8] do not necessarily explore understandings of inequalities holistically from a reciprocal point-of-view (i.e.,understanding the challenges and experiences from the perspective of a person who is facing discrimination). This calls for a more meaningful engagement that would lead to the elimination of internal bias and actionable strategies for an inclusive environment.

In this article, the focus is on the harmful stigma and discrimination experienced by LGBTQIA+ (Lesbian, Gay, Bisexual, Transgender, Queer, Intersex, Asexual Inclusive) people, affecting their physical, mental, emotional, and social well-being and leading to disparities in healthcare access and outcomes [9,10]. To combat this situation, it becomes particularly important for our future medical professionals to develop inclusive attitudes and practices, as they must not only provide excellent healthcare but dismantle the disparities and discrimination that constrain and harm individuals who identify as LGBTQIA+ [11,12].

### Goal of Innovation

Recognizing the growing potential of museum arts for humanity training and critical reflection [13], a program integrating reflective engagement with queer art exhibits was developed. The “Queer Art” program (representation of queer communities through paintings, photographs and other visual media) aimed to help medical students confront and overcome their internal biases, understand multiple perspectives, and improve their confidence in discussing gender inequalities and advocating for inclusivity to address their specific needs.

### Steps Taken for Development and Implementation of Innovation

In this mixed-method evaluation, 24 final-year medical students were randomly selected from the full class of 150 at Panimalar Medical College Hospital & Research Institute, India. Final-year medical students were chosen because of their relatively increased exposure to marginalized and minoritized patients during their clinical rotations.

### Program Implementation

**Figure 1:**
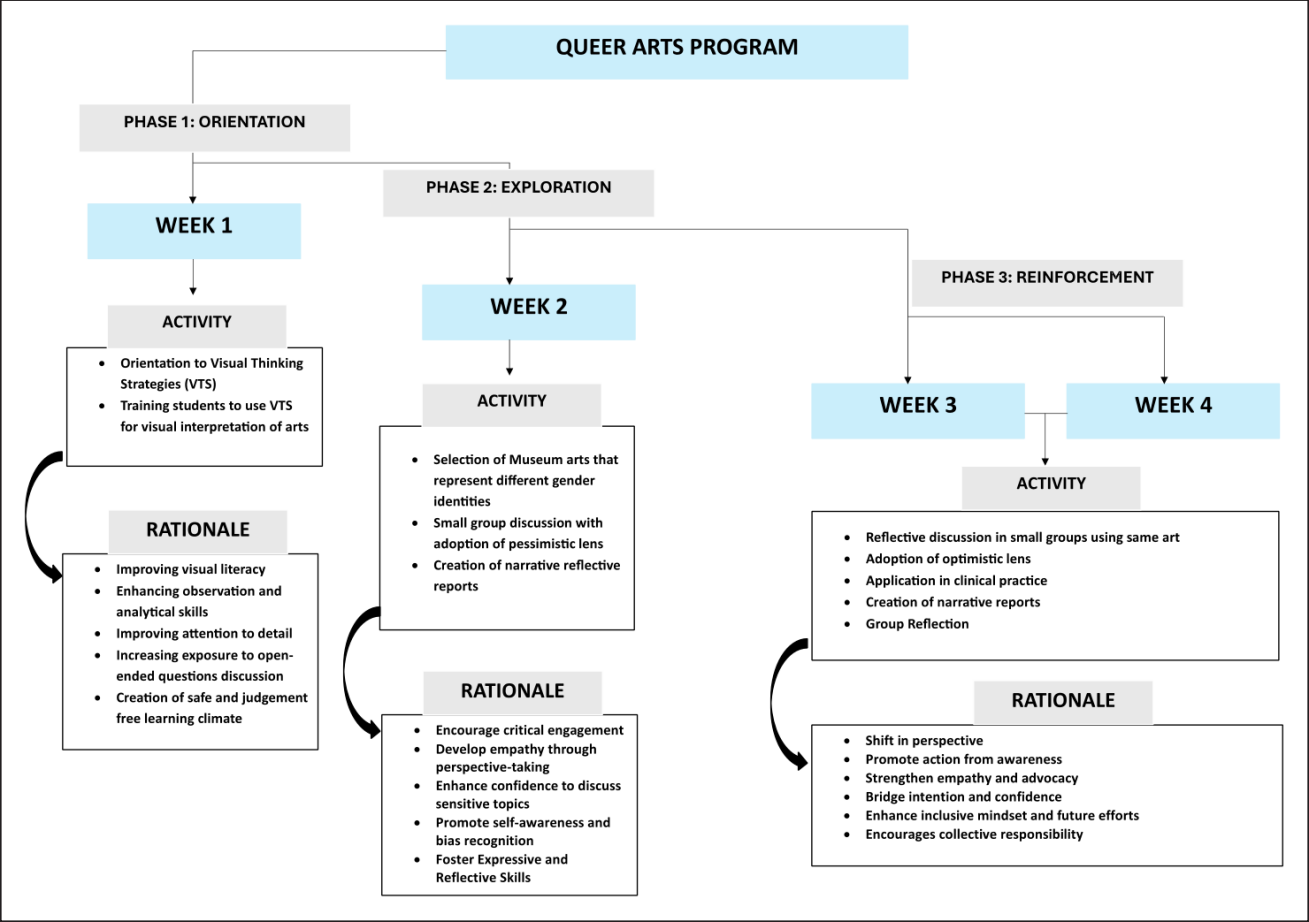
Implementation of Queer Arts Program.

The “Queer Art” program used a 4-week flipped classroom approach across three phases (Figure 1). In phase 1, all students were oriented to the “Visual Thinking Strategies” (VTS), which is a research-oriented and constructivist grounded pedagogical approach used to enhance visual literacy, communication, and critical thinking skills [14].

In phase 2, students in small groups of four were prompted to visit museums or select online museum arts. With facilitator-mediated discussions, students engaged in dialogue, initially adopting a pessimistic lens to explore the artwork. A pessimistic lens in this context refers to analyzing the artwork by focusing on themes of marginalization, oppression, and discrimination faced by the queer community [15]. As an example, while analyzing a historical androgynous painting, students started discussions on how this representation is often erased, ridiculed, or forced into rigid gender norms due to societal stigma. These discussions culminated in the creation of narrative reports by each group, crafted from a queer perspective (as mentioned before the reciprocal point-of-view).

In the third phase, students engaged with the same queer art but from an optimistic point of view. An optimistic lens in this context refers to analyzing the artwork by focusing on themes of resilience, empowerment, acceptance, and progress for the queer community [15]. As an example, on revisiting the historical androgynous painting students now viewed it optimistically discussing how societies must embrace gender diversity, implement inclusive policies, and foster acceptance. Such views were depicted from the physician perspective (a perspective transformation), and synthesized into reports of how they will work to promote inclusivity in their clinical practice.

### Data Collection

For quantitative data, students’ confidence level before and after the program was measured using a 9-item validated questionnaire on a 5-point Likert scale across key aspects such as visual literacy, gender diversity, and gender inequality. The overall effectiveness of the program was evaluated using a 10-item validated questionnaire on a 5-point Likert scale. Both questionnaires were shared online as anonymous surveys.

For qualitative data, in-depth one-on-one semi-structured interviews of 40–45 minutes were conducted after the program to gauge the experience of each student and explore their thoughts and feelings about the art and its implications on their personal and professional perceptions, seeking to understand whether and how this program was effective. Consent was obtained and all interviews were audio recorded. Interpretative Description (ID) was used as a guiding method to analyse the qualitative data [16].

### Reflexivity

To enhance study transparency and credibility, the author engaged in peer debriefings and maintained a reflexive journal to manage personal biases, challenging the initial interpretations and ensuring that the conclusions drawn were rooted in the participants’ actual experiences rather than the author’s preconceptions and hierarchical bias.

### Data Analysis

Both descriptive statistics and paired t-tests were used to compare the confidence level before and after the program. p value of less than 0.05 was deemed statistically significant. Based on these findings, qualitative data were then analyzed to provide deeper insights and context to explain the observed outcomes regarding confidence. This approach allowed the qualitative analysis to focus on specific areas highlighted by the quantitative data, ensuring a more integrated and comprehensive understanding of the results.

ID was employed to systematically analyze the data from a clinical and educational perspective. This approach facilitated the identification of practical implications that could be applied in medical training based on perceptions and experiences of medical students. The first few interviews were open coded to identify initial themes. The codebook was refined and expanded as more data were collected and analyzed. Constant comparative analysis was applied to all transcripts to continuously refine and redefine codes and themes. Analytical memos were written throughout, contributing to the depth of understanding.

### Ethical Approval Statement

This research study received approval from the Institutional Human Ethics Committee at Panimalar Medical College Hospital & Research Institute (PMCHRI-IHEC) (PMCH&RI/IHEC/116; dated 16.02.2024).

### Outcomes of Innovation

A total of 24 final year medical students participated in this training program that included 6 male students and 18 female students. The mean age of the students was 22 ± 1.3.

### Evaluation of Confidence Levels

**Figure 2:**
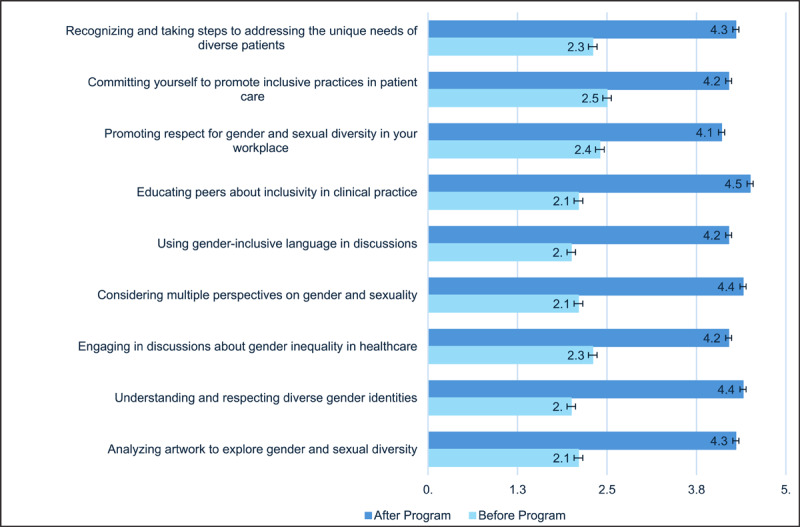
Comparison of confidence levels before and after the training.

On comparing the confidence levels of participants before and after the training with the t-test, there was a highly significant improvement with p-value < 0.0001 across all nine aspects as depicted in Figure 2. Initially, confidence levels were notably lower, particularly in areas such as using gender-inclusive language, educating peers about inclusivity, and analyzing artwork to explore gender diversity. However, after the program, there was a marked improvement across all measured aspects, demonstrating a positive impact on students’ understanding and engagement. Notable changes were observed in fostering respect for gender diversity, recognizing patient diversity needs, and committing to inclusive clinical practices.

### Qualitative Analysis

The subsequent ID analysis explored how the confidence levels of participants were influenced by their perceptions and ideologies prior to the program, compared to the shifts in perspective and confidence observed afterward. This analysis examines how engagement with queer art and reflective activities challenged pre-existing assumptions, fostering a deeper understanding of diversity and inclusion.

#### A. Hesitancy in acting was endemic

This theme explores how hesitancy and pre-conceived thoughts about the LGBTQIA+ community influenced participants’ actions. This was particularly helpful in understanding the low confidence among students before the program about open discussion of gender diversity and understanding multiple perspectives.

##### i) Thoughts about others

Most participants’ greatest concern was a hesitancy to openly discuss gender-related issues. Students also felt that they never had any chance to discuss these sensitive issues in detail: *“I think I have read about inclusivity a few times in the text-books but beyond that I have not really had any discussion regarding it. Many might not be okay with it” [P1]*. This uncertainty about how to approach marginalized individuals and discuss the topic with others could have created a lack of confidence in discussing gender related issues.

##### ii) Power influences change

Part of the students’ hesitancy to act was related to powerlessness when confronted with discrimination, something they were able to identify when engaging with queer arts: *“While engaging in the narrative report synthesis from a queer individual’s perspective, I remembered many incidences where I have seen many transgender women on roads, in buses and trains etc. I have also seen people making faces and instinctively moving to opposite sides. I know it is wrong, but I don’t do anything but watch. It’s a helpless situation”. [P23]* Feeling powerless (in terms of feeling less confident to intervene) in discriminatory situations could have led to the lack of confidence in taking actionable steps, even in future patient encounters. Reflective activities may have helped students identify societal patterns and their own contributions, perhaps boosting confidence in diversity awareness and commitment to change. Discussions about systemic change and institutional accountability helped students see the value of collective action, improving confidence in seeking professional guidance.

A student shared a powerful insight about confidence in acting: *“In our group’s story we incorporated a segment where a medical student genuinely wants to help a transwoman find a job but is unable to do so as he has no influential position. We wanted to bring in this because it’s not enough only when one person wants to bring in a change; the whole society should evolve and most importantly those with the power should change to bring in a change”* [P6] illustrating the potential of authoritative action in healthcare.

#### B. Receptivity increases when emotions are experienced

This theme was derived when students expressed how the queer arts program encouraged them to advocate for LGBTQIA+ individuals and develop that willingness to take actions that was missing before the program.

##### i) Art enhances Connectivity

Students shared that art helped them connect more deeply with gender and sexual diversity. Seeing queer experiences visually represented made them feel more empathetic and aware, helping them grow personally and understand inclusivity in a more meaningful way. A student expressed, *“Apart from what I learn in clinics, creating narratives from the LGBTQ side helped me connect” [P7]*. Many felt that engaging with creative works opened their minds, allowing them to see things from perspectives they hadn’t considered before.

##### ii) Changing lens

Students reflected on how artistic engagement altered their perspectives. One noted, *“Working on the art project, I realized the emotions that LGBTQ individuals often carry, which changed how I view my role as a future doctor” [P16]*. Reflection helped students understand the emotional weight carried by marginalized groups, fostering greater patient respect. Empathy gained through perspective-shifting activities enhanced diversity awareness, building confidence to advocate for inclusivity.

#### C. Inclusivity is not tolerance but acceptance

Finally, students discussed a change in perspective, from tolerance to genuine acceptance. This contributed to greater confidence in fostering a respectful community and promoting inclusive practices for the future.

##### i) You do not have to adjust

Students understood that inclusion means making everyone feel welcomed and more importantly not making significant changes to ‘accommodate’ someone differently. A student commented, “*Some people think it is difficult to have a normal relationship with the LGBTQ community and that they might have to extensively adjust their workplace or educational environment to ‘fit’ them in. This mindset must change, and everyone should understand that inclusivity is not simple adjusting or tolerating someone” [P20]*. Students viewed inclusivity as burdensome and difficult to implement which led to low confidence initially. After the program, students redefined inclusivity as integral to healthcare, building confidence in adopting Inclusive Practices effortlessly and effectively.

##### ii) We are not superior

Students discussed understanding the hierarchical dynamics often found in healthcare, where patients from marginalized communities might feel undervalued, emphasizing the importance of humility and respect in patient care. A student expressed, *“What I have learnt from this training is that nobody is superior here. When we wrote the narrative from a queer community’s point of view, we understood that they are being treated as if they are inferior to us and being “nice” to them is something “noble”. But that’s not true; I believe it is a basic human responsibility. We ought to respect others and accept everyone the way they are”* [P10]. This dialogue encouraged these future healthcare providers to practice medicine from a place of equality.

##### iii) Diversity should enrich not divide

Diversity in healthcare, particularly in understanding and addressing the needs of the LGBTQ+ community has been enriched by broadening students’ perspectives and enhancing their ability to serve a diverse population effectively. A lack of appreciation for diversity limited students’ confidence in engaging with diverse populations. Exposure to diverse perspectives during the program broadened their understanding, enhancing confidence in diversity awareness as reflected by a student. *“In the next part [Reinforcement phase], it was more about how we aim to bring in a change. Diversity in our patient population is not something that will separate us, but it will give us the opportunity to learn and grow together. This mindset is very important in making us better equipped to address all health needs.” [P19]*.

### Evaluation of Program

The evaluation of the program, as shown in Table 1 **(appendix)**, indicates high satisfaction among participants across various dimensions. Notably, the program was particularly effective in helping participants understand the needs of the queer community, reduce personal biases, and change perceptions positively. Overall, the integration of arts into the curriculum was well-received, highlighting its potential to enrich traditional medical training by fostering critical thinking and visual literacy skills.

### Critical Reflection on Your Process

This study aligns with the United Nations Sustainable Development Goals (SGD) 5 (gender equality) and 10, (reducing inequalities), a set of 17 global goals designed to address social, economic, and environmental challenges, aiming for a more just, sustainable, and inclusive world by 2030 [17]. The integration of arts into medical training represents a new yet impactful approach, offering students a unique opportunity for reflective engagement and critical dialogue [18]. Art addresses not just seeing, but also the combination of observation, interpretation, appreciation, exploration, advocation, promotion, communication, and reflection [19]. A growing body of literature highlights the effectiveness of arts-based interventions in enhancing key clinical skills, particularly in observation, communication, and empathy [20,21].

From the analysis of confidence ratings, coupled with qualitative data, initial hesitations in areas such as gender discussion and patient encounters were rooted in fear of judgment, limited exposure, and a lack of structured opportunities to engage with sensitive topics. For many students, societal stigma and internalized apprehension created a sense of passivity or an unwillingness to act, even when they were aware of inequities. These patterns underscore that low confidence was less about ignorance and more about the absence of an environment that encouraged open, reflective dialogue and active engagement with diverse perspectives [22].

A key success to the program was the transformation observed post-program which demonstrates a shift that stemmed not merely from external activities but from deep internal reflection and perspective change. The qualitative responses reveal how students began to question their biases and move beyond surface-level understanding of gender diversity. As they engaged more deeply, they reported a growing ability to empathize, interpret, and navigate complexities related to inclusivity. This shift was not instantaneous but occurred as students reflected on their own discomfort, recognized the limitations of their prior views, and allowed themselves to experience the emotional weight of the narratives they encountered. This process of internal transformation helped students bridge the gap between knowing and understanding, enabling them to see inclusivity as a shared responsibility rather than an abstract concept [23,24].

Arts-based interventions have long enhanced observation and clinical reasoning in medical education, yet this program takes a different approach that ultimately contributes to best clinical practice [25,26]. By engaging with queer art, students moved beyond passive learning to actively confronting issues of inclusivity and social justice. This transformation highlights how confidence, when rooted in reflection and empathy, drives both personal growth and the advancement of more inclusive clinical practice.

The evaluation of the program also highlights its success in fostering meaningful shifts in participants’ perceptions and attitudes toward inclusivity. However, it is important to acknowledge the limitations of this study, including the small sample size and the potential for bias with self-reported confidence levels. Future research could benefit from larger sample sizes and longitudinal follow-up to assess the long-term impact of arts-based interventions on students’ attitudes and behaviors.

The strength of this approach lies in effectively facilitating the discussion among students. A notable challenge faced was initial reluctance among students in openly discussing their personal experiences, even in one-on-one interviews. Further application of this approach may require shifts in educational structures, faculty attitudes, and institutional support systems to create a safe and supportive environment where students feel comfortable sharing their thoughts and feelings without fear of judgment or reprisal [27]. Also, it is important that students recognize and effectively reflect on concrete and detailed aspects of inclusivity rather than broadly discussing stigma and discrimination.

## Conclusion

The program was successful in encouraging medical students to overcome biases and combat discrimination, thus fostering a drive toward an inclusive healthcare workforce. Through reflective engagement with queer museum arts, students deepened their understanding of the complexities of gender and sexual diversity thereby improving their confidence in several critical areas. This process encouraged them to challenge preconceived notions, embrace empathy, and commit to advocating for equity within healthcare. The initiative has not only broadened their perspectives but has also instilled a commitment to fostering an inclusive healthcare environment. By addressing these critical issues through arts-based education, the program has laid a strong foundation for cultivating healthcare professionals dedicated to inclusivity and capable of delivering compassionate, comprehensive care to all patients.

## Appendix

**Table 1:** Evaluation of Program.

EVALUATION OF PROGRAM	MEAN SCORE ± STANDARD DEVIATION

The content of this program aligned well with my overall educational goals in medical training.	4.2 ± 0.9

The creative activities within the program, such as storytelling and art analysis, fostered innovation and helped me think outside the traditional medical education framework.	4.4 ± 0.3

The group activities enhanced my learning experience by providing diverse perspectives and fostering collaborative pragmatic thinking skills.	4.2 ± 0.5

The program helped me better understand the perspectives and needs of the queer community.	4.6 ± 0.2

The program has helped me recognize and reduce my own biases against the queer community	4.7 ± 0.4

I feel more competent in being empathetic towards patients from diverse gender and sexual orientations.	4.0 ± 1.2

The program has significantly improved my visual literacy skills, enabling me to better interpret and understand visual information in the context of gender sensitivities	4.1 ± 0.8

This program has enhanced my critical thinking abilities, particularly in analyzing and responding to complex issues related to gender and sexual diversity.	4.3 ± 1.0

My perceptions of the queer community have positively changed due to the insights gained through this program.	4.7 ± 1.1

The use of arts in our curriculum provided a new educational experience that traditional medical training does not typically offer, enhancing my learning in unique ways.	4.5 ± 0.7
